# Towards an understanding of the behaviour of ruthenium during vitrification of highly active waste: A study of the volatilisation of RuO_2_

**DOI:** 10.1557/s43580-025-01388-4

**Published:** 2025-09-15

**Authors:** Bibi Shehrbano, Colin Boxall, Joshua Turner, Richard Wilbraham

**Affiliations:** 1 Engineering Department, Lancaster University, Lancaster, LA1 4YW UK; 2 United Kingdom National Nuclear Laboratory (UKNNL), Sellafield, Cumbria CA20 1PG UK

## Abstract

Recycling nuclear fuel to recover materials such as uranium and plutonium involves high temperature processes to treat the resulting highly active (HA) waste. This waste contains ruthenium, an important fission product due to its tendency to form volatile compounds and the presence of the radioactive isotope ^106^Ru. These properties, combined with the elevated temperatures during HA waste treatment, necessitate a deeper understanding of ruthenium volatilisation mechanisms. Key ruthenium species present or potentially formed in HA waste include nitro and nitrosyl complexes, and ruthenium dioxide (RuO_2_). This study focuses on the behaviour of RuO_2_ at high temperatures, due to its relevance to the vitrification process used to immobilise HA waste. Preliminary thermogravimetric analysis of commercially available RuO_2_ revealed a dehydration process under N_2_, O_2_, air atmospheres, and volatilisation under oxidising conditions. Post-heating analyses using scanning electron microscopy and X-ray diffraction identified a concurrent, thermally induced recrystallisation process, most prominent under non-oxidising conditions.

**Graphical abstract:**

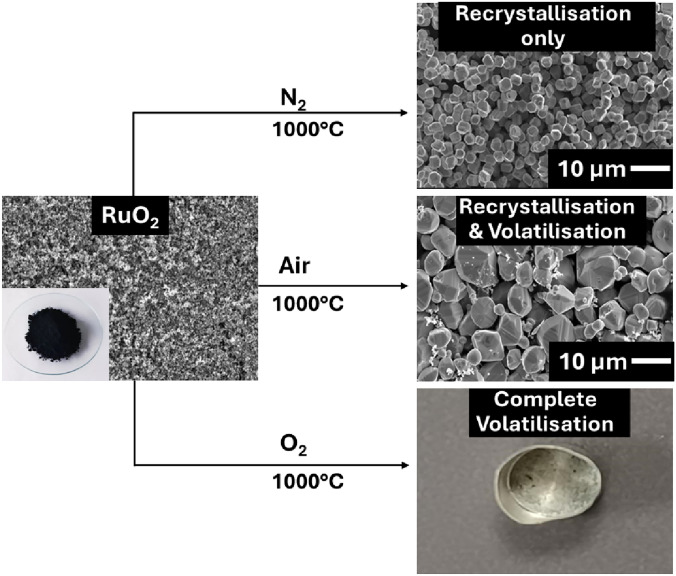

## Introduction

Whilst the Thorp and Magnox reprocessing plants at Sellafield have ended operations [1], there is still highly active (HA) waste awaiting vitrification prior to the decommissioning of these facilities. This HA waste is the byproduct of reprocessing, where U and Pu are separated from the other fission products that are within spent nuclear fuel (SNF) [2]. At Sellafield, such reprocessing involves an initial dissolution of the spent fuel in nitric acid before it is fed into the PUREX (Plutonium–Uranium Reduction Extraction) solvent extraction process [2, 3]. The first stage of PUREX results in the extraction of the U and Pu (and some Np) into an organic solvent phase, leaving all other fission and activation products in the nitric acid phase. The latter is referred to as Highly Active Liquor (HAL) [4, 5]. Treated as a waste, the HAL is concentrated through evaporation at the Highly Active Liquor Evaporation and Storage (HALES) facility and then stored in Highly Active Storage Tanks (HASTs) before being transferred to the Waste Vitrification Plant (WVP). Here it is first calcined to produce a compositionally complex calcine product which is then vitrified for long-term containment [6].

**Fig. 1:**
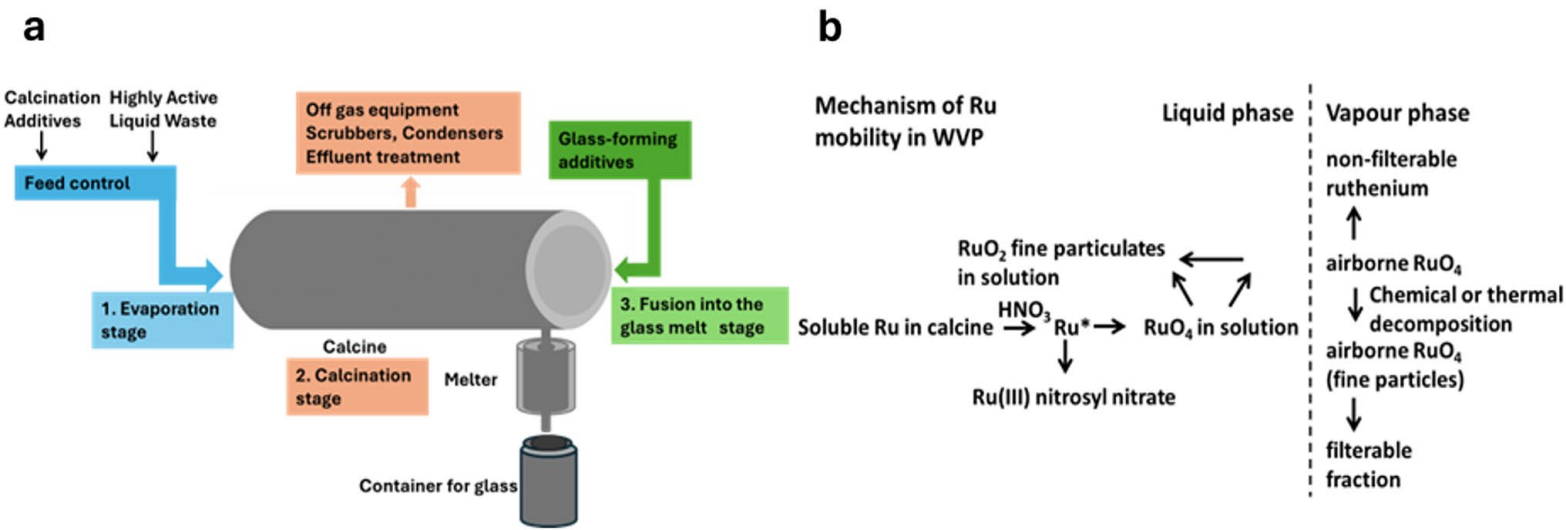
**a** Schematic of the HLW vitrification process on the Sellafield site [5], **b** mechanisms of ruthenium mobility in WVP [6]

Due to the high temperatures involved in calcination and vitrification, it is important to understand the behaviour of thermally volatile radionuclides in order to maintain process control and prevent unwanted releases. Ruthenium, which can form volatile compounds at elevated temperatures and that possesses an isotope of high specific radioactivity (^106^Ru, t_1/2_ = 365 days), is one such radionuclide. There are three stages to the vitrification process where potential volatilisation of ruthenium can occur: *evaporation* of HAL to dryness, the *calcination* of the solid residue, and the *fusion* of the calcined oxides into a glass [7]. If ruthenium in the HAL can transform into volatile compounds during the vitrification process, those compounds may then enter the process off-gas system (Fig. 1a) [5, 7, 8]. At the start of the evaporation stage of the vitrification process, where temperatures are relatively low, the extent of Ru volatilisation is also very low, whereas at the higher temperatures achieved at the end of the evaporation and the beginning of the calcination stage, higher extents of ruthenium volatility have been observed [7]. The extent of this may be dependent upon a number of process parameters, including calciner atmosphere composition and Ru:NO_3_^−^ ratio. In order to suppress volatilisation during the evaporation and calcination stages, sugar is added to the calciner as a reducing agent [7]. However, care is required during this addition as overdosing may lead to blockages in the off-gas system [5, 8].

Ruthenium exists in a number of forms in the nitric acid media that comprises the HAL, predominantly as nitrosyl and nitro complexes, Ru(NO)^3+^ and Ru(NO_2_)^2+^ [9, 10]. The thermodynamics and kinetics of the volatilisation of ruthenium in the WVP may vary considerably depending on its initial speciation within the HAL. However, during the calcination step, the nitro and nitrosyl complexes of Ru(III) within the HAL may be oxidised to RuO_2_, which may also have an opportunity to volatilise during vitrification (Fig. 1b).

There have been numerous studies on the volatilisation behaviour of nitro/nitrosyl ruthenium and RuO_2_ under high temperature conditions (> 1000 °C) in the context of nuclear accidents. Holdoway investigated ruthenium volatilisation and RuO_2_ deposition by heating ruthenium dioxide to 1050 °C in an O_2_ stream, with the subsequently vaporised RuO_2_ then being progressively deposited along the stainless-steel thermal gradient tube. The results obtained indicated the existence of volatile ruthenium compounds, most probably ruthenium tetroxide, RuO_4_ [11]. Schäfer et al. have conducted equilibrium measurements on the ruthenium-oxygen system and found that at temperatures ≥ 1200 °C, Ru volatilises primarily as  RuO_3_. In contrast, at temperatures lower than that, RuO_4_ becomes the dominant species. Ohnet et al*.* [13–15] also investigated the behaviour of ruthenium under oxidative conditions within the stainless tubes of a PWR cooling system under accident scenarios. Using a maximum temperature of 1200 °C in steam/air mixtures, their results showed that a significant part of the volatilised ruthenium, up to 95%, is deposited along the tube, with the remaining part being transported almost in a gaseous form, attributed to RuO_4_. Kajan also studied the effect of nitrogen oxides and nitric acid on the transport of ruthenium through the primary circuit of a nuclear power plant. In their study, ruthenium dioxide powder was heated to 1027, 1227, and 1427 °C in an oxidising flow, producing 13.9, 13.9, and 20.2% gaseous ruthenium oxides, respectively [4]. Complementary to these accident-focussed studies, Lawson et al*.* have studied the volatilisation potential for ruthenium at various stages of the vitrification process itself using a HAL simulant solution. They found that very low levels of volatilisation occurred during the evaporation stage due to the relatively low temperatures employed. In contrast, at the calcination stage, 75% of the total Ru content of the HAL simulant was lost under the associated higher temperature conditions [7].

However, studies conducted at temperatures below 1000 °C, such as that by Lawson et al., are less common. Under such conditions and based on the work of Schäfer et al., RuO_4_ would be expected to be the main product of the volatilisation of RuO_2_ in oxidising atmospheres [12]. Therefore, our focus has been to understand the role of RuO_2_ in the generation of volatile ruthenium at these lower temperatures, such that the resultant data may help enable more efficient operations during maintenance and facility downtime [6, 16]. As mentioned above, this study focussed on the behaviour of ruthenium during the vitrification (where the fission products, after their separation from the dissolved fuel, are put into a glass) of highly active liquor (HAL). However, it may also be of utility at the headend or a reprocessing plant (where the spent fuel is dismantled and dissolved in near-boiling nitric acid). Both the headend and vitrification plants include processing stages where Ru-bearing solutions of nitric acid are exposed to temperatures of at least 150 °C, and where, unexpectedly in light of the above, Ru has been seen to volatilise in the plant [6]. This unexpected low temperature volatilisation complicates the control and abatement of ruthenium during reprocessing and vitrification operations, highlighting the need for a deeper understanding of its behaviour under these conditions.

Thus, this study focuses on the structural and morphological transformations that may occur during thermal treatment of RuO_2_ as a putative immediate precursor to the formation of more volatile higher oxides. As alluded to above, the study also focuses on these transformations under relatively low temperature (< 1000 °C) conditions but under a range of gas atmospheres, including non-oxidising nitrogen and oxidising air and pure oxygen atmospheres. The resultant insights into these transformations will provide a foundation for future investigations into the kinetics of the volatilisation process itself and the potential inhibiting or accelerating effects on that process of other fission product species present in the calcine matrix.

## Methods and materials

RuO_2_ powder was of AnalaR grade and was supplied by Sigma-Aldrich (Gillingham, Dorset, UK). Scanning Electron Microscopy (SEM) images were obtained using a JEOL 6010-LV at magnifications of ×1000 and ×2000 to study the surface morphology of RuO_2_. Examinations were conducted at 20 keV using secondary electron imaging (SEI) and a magnification of 1000×.

X-ray diffraction data sets were recorded at room temperature using a Rigaku Smartlab X-ray diffractometer. These measurements employed a parallel beam configuration with two Ge monochromators (220) and a Cu X-ray target, operating at 45 kV and 200 mA, with a Kα wavelength of 1.540593 Å. The X-ray Diffraction Analysis (XRD) scans of RuO_2_ samples, conducted before and after ThermoGravimetric Analysis (TGA) involved 2θ scans at a rate of 2° per minute, with step intervals of 0.01°. A 5 mm exit slit was used alongside a Dtex250 Ultra 1D detector, ensuring that the beam illuminated the entire sample.

**Fig. 2:**
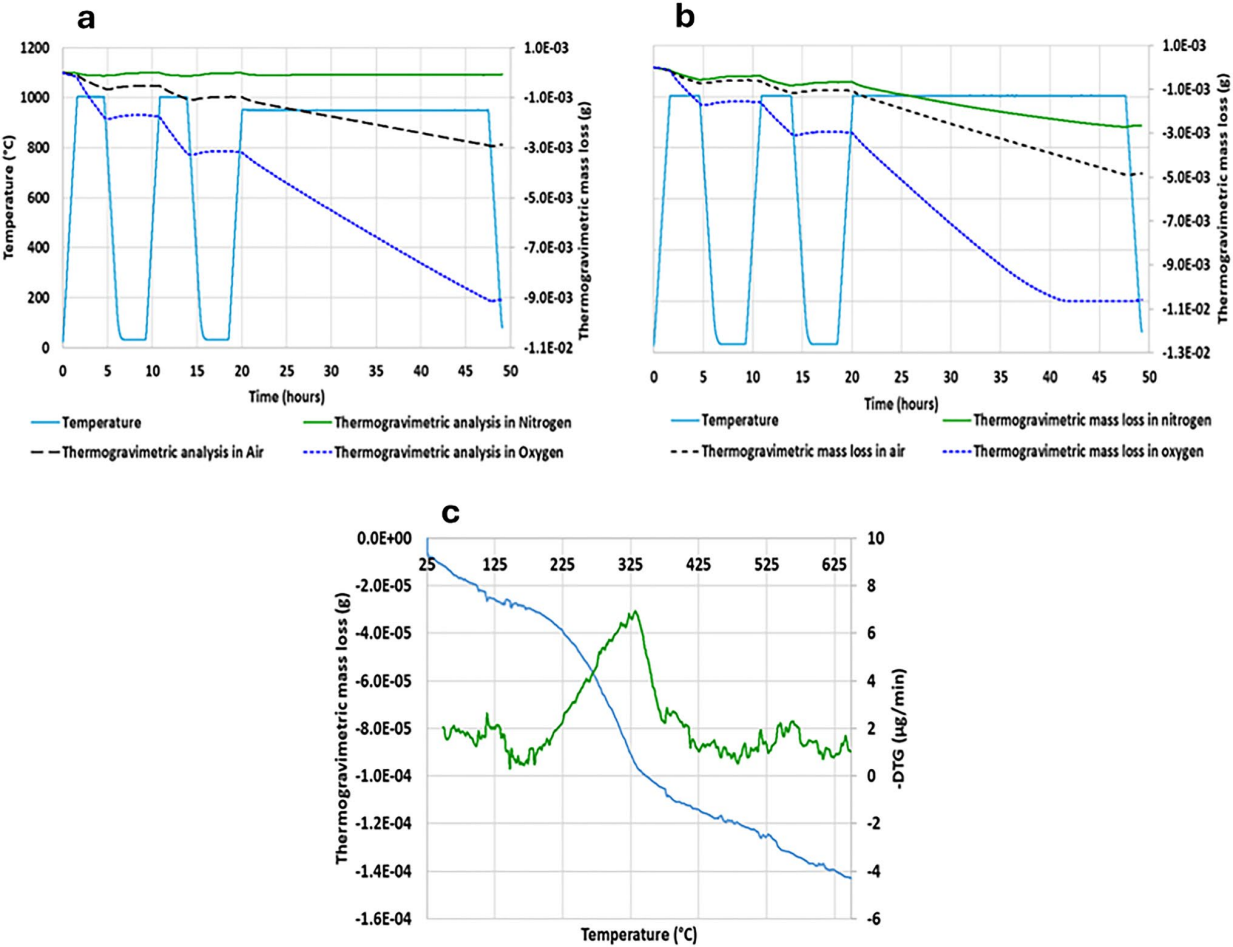
**a** Thermograph of heating RuO_2_ samples under nitrogen, air and oxygen, at a dwell time temperature of 950 °C; and **b** RuO_2_ samples under nitrogen air and oxygen at a dwell time temperature of 1000 °C; **c** TG and DTG analyses of RuO_2_ ramped between 25 and 625 °C during the first dehydration cycle under air, TG data taken from Fig. 2b

TGA data were collected using a Hitachi STA7200 model under conditions of 0.3 L/min of nitrogen, air, and oxygen flow, with approximately 10 mg of RuO_2._ The TGA thermographs were acquired for the thermogravimetric mass loss of RuO_2_. Unless stated otherwise, samples of approximately 10 mg were heated at a constant rate of 10 °C/min, and continuous mass loss was observed up to 1000 °C in the TGA analysis. The heating profile shown in Fig. 2a and b involved three heating/cooling cycles. In the first and second cycles, the temperature was ramped up to 1000 °C, held for 3 h, and then cooled back to room temperature. In the third and final cycle, the samples were subjected to thermal treatment at 950 and 1000 °C as well as an extended dwell time of 27.5 h.

## Results and discussion

Figure 2a and b shows TGA traces recorded for RuO_2_ samples under N_2_, air and O_2_ atmospheres using the three thermal cycle approach described above with third cycle hold temperatures of 950 and 1000 °C, respectively. Cognisant that the as-received RuO_2_ may have a layer of physisorbed water present (vide infra), the first two thermal cycles were performed to ensure that the as-received samples had been fully dehydrated before extended dwell period studies of a third cycle. During the third cycle dwell time, the following percent mass losses were observed: under O_2_, 100 and 98.6% at 1000 and 950 °C, respectively; under air, 48 and 30% at 1000 and 950 °C, respectively; under N_2_, 10 and 0.9% at 1000 and 950 °C, respectively.

The presence of oxygen during the TGA runs conducted under air and pure O_2_ atmospheres provides a mechanism for RuO_2_ oxidation to volatile higher oxidation state ruthenium oxide species such as RuO_4_, as suggested by Mun et al. and Lawson et al. [6, 7]. These are thermodynamically unstable and decompose on cooling to form anhydrous RuO_2_ crystals [6].This post-volatilisation behaviour notwithstanding, the availability of this oxygen-enabled route from RuO_2_ to volatile higher oxides provides an explanation for the mass losses observed during the high temperature dwell periods under air and pure O_2_ atmospheres in Fig. 2a and b. Faster rates of mass loss are observed under the pure O_2_ compared to the air atmosphere, suggesting, not unexpectedly, that O_2_ concentration plays a role in the kinetics of RuO_2_ volatilisation.

However, this does not explain the (smaller) mass losses observed in Fig. 2a and b during the high temperature dwell periods of the TGA runs conducted under pure N_2_, suggesting that sample dehydration also plays a minor, albeit small, role in the mass losses observed during all TGA runs of Fig. 2a and b.

This hypothesis is further supported by closer examination of the thermogravimetric (TG) analysis data and corresponding derivative thermogravimetric (DTG) analysis obtained from a RuO_2_ sample heated at a rate of 10 °C/min from room temperature to 1000 °C in an air atmosphere. As shown in Fig. 2c, a continuous mass loss is observed up to 625 °C, resulting in a total mass loss of 13% by the end of the scan. Liang et al. observed a similar mass loss of ~ 12% during TG analysis when heating RuO_2_ samples from room temperature to 330 °C [17]. An observation that they attribute to the presence of RuO_2_·H_2_O in their sample. Similar to the DTG data of Fig. 2c, DTG analysis of their mass loss data revealed two broad peaks at ~ 100 °C and ~ 330 °C, the former of which they attribute to the loss of physisorbed water. The latter they suggest may be due to either a phase transition to a crystalline state or the loss of chemisorbed water bound in the form of RuO_2_·H_2_O. Given the mass loss observed in the TGA data, they suggest that as the analysis temperature increases above 100 °C, RuO_2_·H_2_O undergoes dehydration to form RuO_2_·xH_2_O, where x is either zero or less than or equal to 1. Above 330 °C, anhydrous RuO_2_ is fully formed [17, 18]. Given the similarity of their data to that of Fig. 2c, it is not unreasonable to conclude that similar dehydration processes are occurring in the TGA data of Fig. 2 and especially that recorded under pure N_2_.

**Fig. 3:**
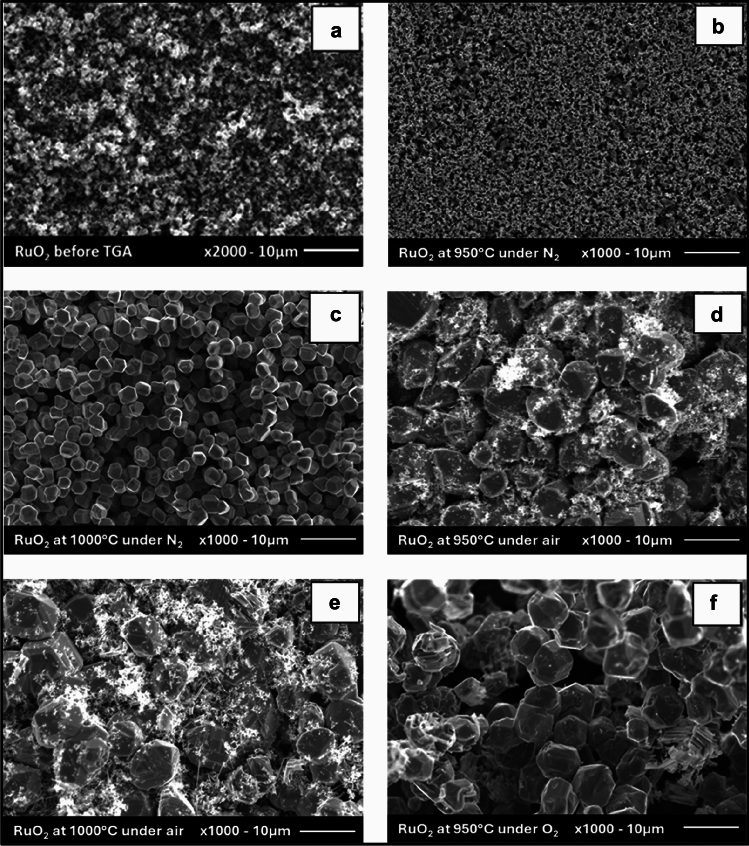
**a** SEM image of RuO_2_ before thermal treatment. **b** SEM image of RuO_2_ after thermal treatment at dwell time temperatures of 950 °C and **c** 1000 °C in nitrogen. **d** SEM image of RuO_2_ after thermal treatment at dwell time temperatures of 950 °C and **e** 1000 °C in air. **f** SEM image of RuO_2_ after thermal treatment at dwell time temperature of 950 °C in oxygen

The morphology of the RuO_2_ was examined by SEM before and after TG analysis, Fig. 3. Lennar et al. [18] identified two main forms of RuO_2_: anhydrous and hydrated, suggesting that hydrated RuO_2_ exhibits a granular morphology. As shown in Fig. 3f, the as-received RuO_2_ used in the studies presented here was granular, which, in addition to the TGA data, suggests it was in a hydrated form. Figure 3a, c, and e shows the changes in the morphology of the RuO_2_ samples after thermal treatment at dwell temperatures of 950 °C in N_2_, air and O_2_ atmospheres, respectively.

Under all atmospheres studied, the thermal treatment causes the sample particle size to grow and, in the presence of oxygen at this temperature, recrystallise as larger crystals with a rutile crystal-like morphology, Fig. 3(a), consistent with anhydrous RuO_2_ as described by Lennar et al. [19]. In contrast, after thermal treatment at 950 °C under nitrogen, Fig. 3(b), the particles appear significantly smaller compared to those treated in air or oxygen. Under all atmospheres after thermal treatment at 950 °C, the recrystallisation process appears incomplete, with some residual powder still present. However, after treatment under nitrogen at the higher dwell temperature of 1000 °C, fully formed rutile crystals were achieved (Fig. 3c), indicating that the sample was fully dehydrated (as per Lennar et al., *vide supra*). After analogous thermal treatment in air, crystalline structures are observed alongside residual powder, Fig. 3(e), which may consist of unrecrystallised RuO_2_ or fragmented crystals. In contrast, under an oxygen atmosphere at 1000 °C, complete volatilisation of ruthenium is observed, indicating total thermal decomposition. Taken as a whole, Fig. 3 suggests that two parallel processes are in operation during thermal treatment of RuO_2_: (i) a dehydration accompanied by a recrystallisation to form anhydrous rutile RuO_2_; and (ii) an oxidation to produce volatile higher oxides of Ru. At 950 °C, process (i) appears to be faster than process (ii). However, both are accelerated by increasing temperature, process (ii) more than process (i), so that at 1000 °C, process (ii) is no longer faster than process (i).

**Fig. 4:**
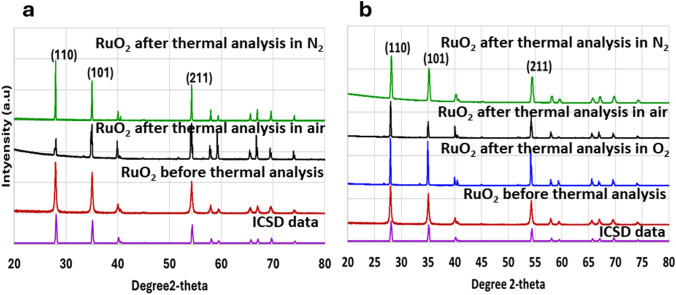
**a** XRD analysis of RuO_2_ samples before and after TGA runs under N_2_ and air at a dwell time temperature of 1000 °C, and **b** RuO_2_ samples under N_2_, air and O_2_ at a dwell time temperature of 950 °C

In order to explore whether a rutile structure does indeed obtain XRD patterns, Fig. 4, were recorded for all samples in Fig. 3 for 2θ = 20–80°, i.e., both before and after all thermal treatments of Fig. 2—the exception being the RuO_2_ sample held at 1000 °C under O_2_ where full volatilisation of the sample was observed. Figure 4 also shows the ICSD reference for the rutile crystal structure RuO_2_ (ICSD 15071: 28.10°, 35.10°, and 54.40°). A comparison of the sample XRD of Fig. 4 with this reference indicates that all samples studied have the rutile structure, consistent with the structure inferred from the crystal morphologies seen in the SEM images of Fig. 3. The XRD pattern of the thermally treated RuO_2_ samples generally exhibit more well-defined, sharper peaks compared to the broader and less intense peaks observed in the hydrous untreated RuO_2_ samples. This suggests a thermally induced transition to a more crystalline structure, again as observed in the SEM images of Fig. 3. These sharper peaks also suggest the formation of larger grain sizes in the thermally treated samples [20], again consistent with Fig. 3.

RuO_2_ crystal sizes were calculated using the Scherrer equation [21]. Before the thermal treatment, the RuO_2_ crystal size was found to be 514.32 nm; however, after the thermal cycle under N_2_, the crystal size increased to 1926 nm at a dwell time temperature of 1000 °C. A slight size decrease to 423 nm was observed at a dwell time temperature of 950 °C, although this is likely due to experimental error. This is consistent with the SEM images recorded after similar thermal treatments under nitrogen: at 1000 °C significant recrystallisation into larger rutile particles is observed, with very little change in sample morphology being seen at the lower temperature of 950 °C. For analogous data recorded after thermal cycles under air, the crystallite sizes were found to be 1845 and 1621 nm for dwell time temperatures of 950 and 1000 °C, respectively. For analogous data recorded after thermal cycles under oxygen, the crystallite size was found to be 2281 nm for dwell time temperatures of 950 °C; all RuO_2_ had volatilised over the timescale of the experiment at 1000 °C, and thus there was no sample to analyse, respectively. This shows that thermal treatment increases the crystal size of RuO_2_, with higher dwell time temperatures generally promoting higher degrees of crystallite growth. The atmosphere during treatment also plays a crucial role, with oxygen-rich environments (air and O_2_) yielding larger crystal sizes compared to inert N_2_, particularly at 1000 °C (Fig. 4).

## Conclusions

This study provides a combined Thermogravimetric Analysis (TGA), X-ray Diffraction (XRD) and, Scanning Electron Microscopy (SEM) study of the effects of cycling hydrated RuO_2_ samples between room temperature and 950 and 1000 °C under nitrogen, air and oxygen atmosphere. It was observed that, during thermal cycling of the material, three processes occur as follows:

1. Dehydration of the as-received hydrated RuO_2_ via two processes occurring at 100 and ~ 330 °C, in agreement with previously published data [16]. These processes were attributed to the loss of physisorbed water at 100 °C and the loss of chemisorbed water at  ~ 330 °C.
2. Oxidative conversion of RuO_2_ to a volatile higher oxide, either RuO_3_ or, more likely in the sub-1000 °C temperature ranges employed in this study, RuO_4_ in air or O_2_. Formation of higher oxides was not observed in N_2_.
3. A recrystallisation process during which the as-received material recrystalises into larger crystallites consistent with a rutile crystal structure, confirmed by XRD measurements. The SEM-observable recrystallisation is most noticeable during thermal holds at 1000 °C under a nitrogen atmosphere, with the extent of crystal growth being seen to increase with hold temperature and oxygen concentration in the atmosphere used during the thermal treatments (pure nitrogen, air, pure oxygen).

Taken as a whole, these observations suggest that two parallel processes are in operation during thermal treatment of RuO_2_: (i) a dehydration accompanied by a recrystallisation to form anhydrous rutile RuO_2_; and (ii) an oxidation reaction to produce volatile higher oxides of ruthenium. At 950 °C, process (i) appears to be faster than process (ii). However, both are accelerated by increasing temperature, process (ii) more than process (i) so that at 1000 °C, process (ii) is no faster than process (i).

In conclusion, this study demonstrates the significant influence of temperature and atmospheric conditions on the phase composition and crystallisation. The findings highlight the complex interactions between RuO_2_ and its environment, offering new insights into its structural transformations during thermal treatment. These findings will serve as a robust foundation on which can be built a study of the potential volatilisation of RuO_2_ at temperatures in the range 440–800 °C, the lower end of which encompasses temperatures at which calcination occurs during the HAL vitrification process.

## Data Availability

The datasets generated during and/or analysed during the current study are available from the corresponding author on reasonable request.
